# A call to action to address rural mental health disparities

**DOI:** 10.1017/cts.2020.42

**Published:** 2020-05-04

**Authors:** Dawn A. Morales, Crystal L. Barksdale, Andrea C. Beckel-Mitchener

**Affiliations:** Office for Disparities Research and Workforce Diversity, National Institute of Mental Health, Bethesda, MD, USA

**Keywords:** Mental health, health disparities, suicide, services research, behavioral health care, provider shortages

## Abstract

Rural residents in the USA experience significant disparities in mental health outcomes even though the prevalence of mental illness in rural and metropolitan areas is similar. This is a persistent problem that requires innovative approaches to resolve. Adopting and appropriately modifying the National Institute on Minority Health and Health Disparities research framework are the potential approaches to understanding how these disparities might be addressed through research. Using this research framework can facilitate interrogation of multiple levels of influence, encompassing complex domains of influence and consideration of the entire life course trajectory, which is consistent with several National Institute of Mental Health priorities.

## Introduction

Approximately one-fifth of the US population live in a rural area, and about one-fifth of those living in rural areas, or about 6.5 million individuals, have a mental illness [1,2]. Though the prevalence of serious mental illness and most psychiatric disorders is similar between US adults living in rural and urban areas [3,4], adults residing in rural geographic locations receive mental health treatment less frequently and often with providers with less specialized training, when compared to those residing in metropolitan locations. The reasons underlying this mental health treatment disparity are well documented and include reduced access to providers and limited availability of specialty mental health care in rural areas, lack of trained mental health providers and care coordination in rural medical care, and underutilization of available services [5,6]. In addition, the uptake of innovative approaches to mental health care has not been as consistent in rural areas as it has in metropolitan areas, thus exacerbating already wide differences in access and quality of care [7].

**Fig. 1.**
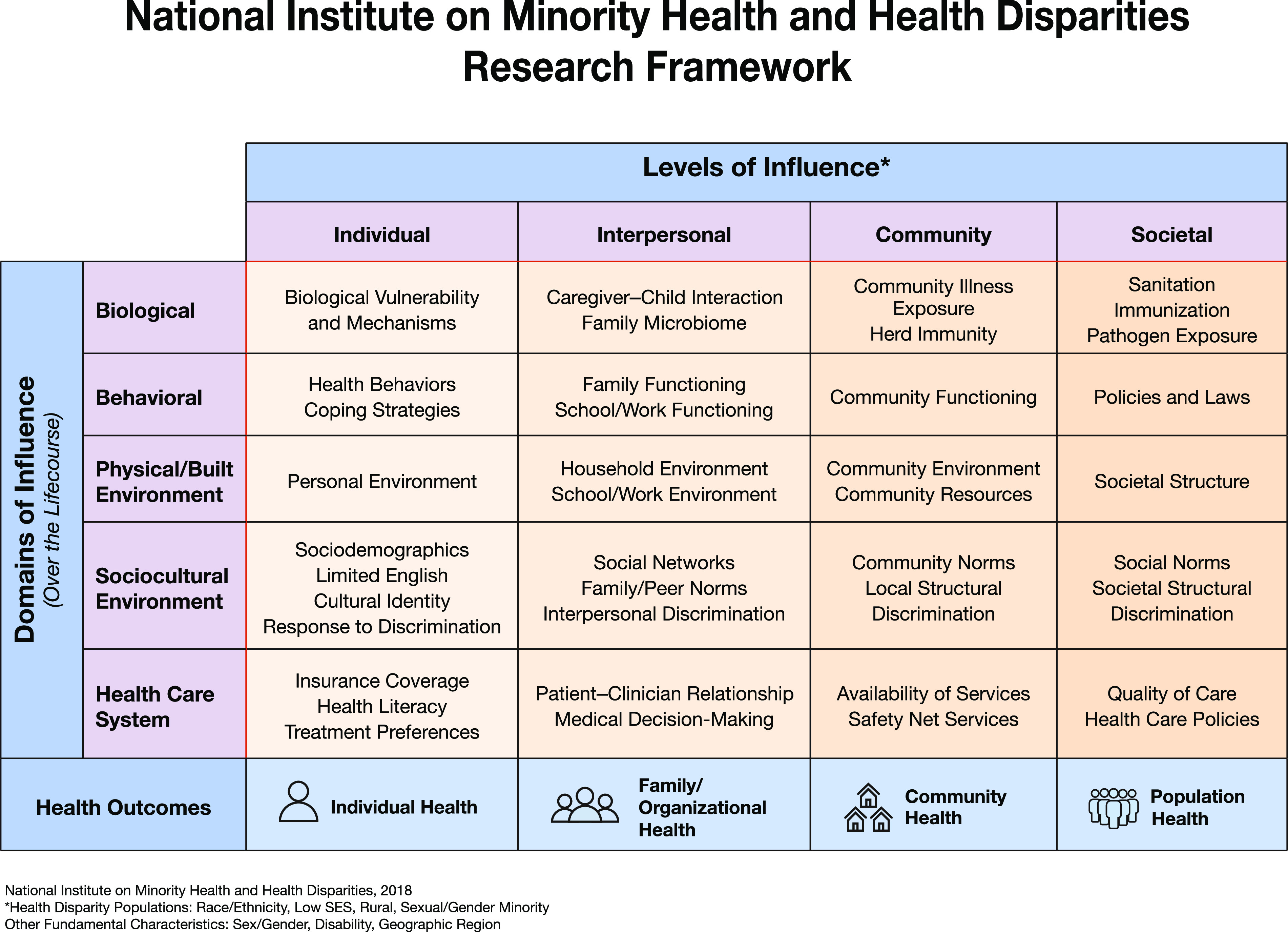
This framework can be used or adapted to address rural mental health disparities [11].

In this paper, we highlight the National Institute on Minority Health and Health Disparities (NIMHD) research framework [8] (see Fig. 1) as a way to conceptualize the complexity of rural mental health disparities and to enhance study designs that advance the rural mental health research agenda. The NIMHD research framework is especially relevant for clinical and translational scientists, especially those in mental health who are focused on translating research findings into culturally and linguistically appropriate interventions and tools that reduce health disparities, improve minority health and quality of life, and ultimately improve health equity [9] in rural settings. More specifically, the NIMHD framework is relevant to mental health research because it addresses multiple dimensions that influence (mental) health outcomes, at multiple levels, within a developmental and a multilevel context. Much the way NIMH’s Research Domain Criteria is a research framework for investigating mental disorders, the NIMHD framework can be applied to understand the nature of mental health disparities and how to address them. The NIMHD research framework characterizes health disparities in a grid-like representation with domains of influence along the rows (biological, behavioral, physical/built environment, sociocultural environment, and healthcare system) and levels of influence [10] along the columns (individual, interpersonal, community, and societal), with a bidirectional life course arrow cutting across the domains of influence column to indicate the influence of early adverse events, chronic and cumulative exposures, intergenerational transmission of both risk and resilience, as well as critical periods in development. The challenge in resolving the disparity in rural mental health outcomes lies in part in the fact that so many rural residents and families exist at a point of dimensional overlap often different from their metropolitan counterparts. We use the general term *point of dimensional overlap* to describe where individuals (and other levels of influence, see Fig. 1) are situated, at the intersections of the many dimensions from the research framework. Rural residents in large part experience substantial disparity in mental health outcomes because their points of dimensional overlap are associated with higher risk for poor mental health outcomes. Rural residents are more likely to be at points of dimensional overlap that include challenges accessing care systems due to geographic isolation, reduced access to and engagement with appropriate providers, lower socioeconomic status, generally lower levels of educational attainment, as well as reluctance to seek help due to discrimination and stigma [3–6]. Dimensional overlap is distinct from interaction in that the various dimensions may combine in a null, additive, or multiplicative fashion for various levels of analysis (e.g., individuals, families, and communities). Thus, through this lens, rural mental health disparities can be attributed to the complex influence of multiple domains, levels, and life course events that combine to create remarkably difficult and persistent health challenges that individuals who live in rural settings may face.

## Dimensions in Rural Mental Health Disparities Requiring Special Consideration

To illuminate the complexities of rural health disparities and the initial application of NIMHD research framework, we present a brief hypothetical example that illustrates the idea of dimensional overlap when considering the challenges of rural residents. Consider “Alex,” a transgender woman with a mental illness, who resides in a rural community. Alex needs to see a psychiatrist once a month for medication management, and a psychologist or counselor every other week for help with coping and symptom management, and telemedicine options are not yet available in her community. Although Alex works full-time and has medical insurance through her employer, there are no mental health professionals nearby because of provider shortages that are common in rural areas. Alex needs to drive even further than other residents of her rural community since she needs mental health providers who are accepting and knowledgeable about care for transgender individuals. To attend appointments, Alex will have to make multiple trips because her insurance does not permit two behavioral health appointments in one day, a common restriction in the USA, but the effect of this is compounded by the fact that she must travel long distances for scheduled appointments. Additionally, since Alex lives and works in a rural area, her efforts to get care are also complicated by the fact that her workplace insurance may be exempt from covering mental health benefits or may impose benefit limitations for mental health services. There may be additional employer or coverage policies as well as legal constraints that add barriers to accessing necessary care [12]. This concerns Alex since her employer is unaware of her mental health history or her sexual/gender minority status, and Alex is worried about stigmatization and discrimination she may face related to both if her employer or anyone in her community were to find out. Due to the smaller size of Alex’s community, her absences will be noticed, which adds to her stress. Alex’s path to wellness is affected by multiple dimensions which each likely contribute to risk of poor health outcomes, some of which may combine in a more-than-additive fashion. Therefore, using this example, a researcher may focus on how domains of influence (Fig. 1) such as cultural identity (sociocultural environment/individual influence), insurance coverage (health care system/individual influence), and community norms (sociocultural environment/community influence) combine to exacerbate mental health disparities in rural places, and what interventions can be developed to reduce them. It is critical that researchers in rural mental health consider multiple domains and levels of influence in their study designs and address the complexities of the multidimensional risks and protective factors in rural settings.

### Mental Health Providers

There is a significant lack of access to specialty mental health care in rural areas in the USA [5]. It is estimated that as many as 65% of nonmetropolitan counties do not have psychiatrists [6], and over 60% of rural Americans live in designated mental health provider shortage areas [13]. Specialty mental health services are scarce in rural areas, which is likely to contribute to these disparities in care [14].

Historically, the primary strategies used to address the lack of access to specialty mental health care in rural areas have been to provide economic incentives and training in rural settings [15]. For example, the National Health Service Corps (NHSC) was created in 1970 to provide primary health care clinicians for the underserved [16]. It is estimated that the NHSC has provided more than 1100 mental and behavioral health professionals to rural settings across the USA, and that one in three NHSC clinicians provides behavioral health care in high-need areas [17]. While these efforts are laudable and help build the behavioral health safety net and service capacity for underserved populations, the outcomes are variable. A retrospective analysis of NHSC alumni showed that while 60% of alumni are in service to the underserved up to 15 years after their service is completed, only about half stayed close to their original service site [16]. One lingering question is that of selection effects, since it is unclear whether this program simply selects for providers who would have worked in underserved areas anyway [18]. Still, the data show that a significant number of providers do not remain in a rural setting, which challenges the patient–provider relationship and continuity of care.

Another common strategy is to increase the numbers of rural residents earning provider credentials and gaining licensure for practice in rural areas. The idea is that people who grow up in rural areas may seek training elsewhere but might want to return home to live and practice. Some state governments developed local programs with financial incentives for health care providers and this effort peaked in the 1990s [19] but the impact has not been rigorously evaluated. Continued efforts have been made to address primary care practitioner training for mental health care, as well as developing and validating novel provider roles and innovative practice arrangements. In particular, there have been efforts to establish professional provider categories (e.g., peer worker, behavioral health aides, and navigators) in mental health that can provide essential services in rural areas. The most well-developed model of this type of effort is the behavioral health aides [20] in Alaskan Native (AN) settings in Alaska.

Aside from the lack of trained and certified providers, there are other contextual and cultural factors which shape disparities in mental health treatment in rural populations. While there may be no uniform definition of rurality, there are several commonly accepted factors that shape the culture of rurality and its impact on mental health, such as low population density and remoteness, isolation, poverty, religion, social support, and stigma [21,22]. There is variability across these factors, but their combined influence on mental health care remains significant. Research suggests that individuals living in rural areas are less likely than residents of urban areas to seek professional help for psychological distress for several reasons including stigma (both public and self-directed) and limited mental health literacy [14,22–24]. Contextual, social, and socioeconomic factors such as lack of transportation, higher poverty rates, and varied insurance coverage [25] are also critical in limiting individual’s access to treatment and utilization of professional services.

A Success Story for Rural Places

The RAISE (Recovery After an Initial Schizophrenia Episode [26]) early treatment program for first episode psychosis was designed to be implemented in diverse community clinics, with flexibility built in to adapt to places with a wide range of patient flows and resources. NIMH-funded research trained over 200 community mental health professionals, including some from rural areas, who each designed, staffed, and implemented a successful intervention program. The manuals and procedures from this project are widely available, allowing other community clinics to adopt this highly effective best practice.

### Age

Although the reported rates of mental health issues are comparable for rural and metropolitan residents, rural children from small communities are more likely to have mental, behavioral, and developmental disorders than children living in cities and suburbs [27]. Despite this, in rural communities, there are significant shortages of trained mental health specialists able to provide quality care to children and adolescents in need [27]. Poverty and perinatal or early childhood teratogen exposure are possible causes for this disparity, since one in four rural children live in poverty compared to one in five children nationally [27]. Similarly, there is also an urgent need for mental health services for rural older adults. It is estimated that approximately 10–25% of rural older adults had diagnosable psychiatric illnesses, which require specialty geriatric mental health services, many of which are not available in rural communities [28,29].

The overall gap in suicide rates between urban and rural areas has grown steadily since about 1999 [30] and rural suicide rates are currently almost double those of metropolitan areas for both males and females [31]. Two age groups particularly affected are early adolescents aged 10–14 and adults aged 25–34, with suicide rates almost four times higher than for adults older than 34 years [30]. Service accessibility, availability, lack of insurance with mental health benefits, access to lethal means, and both geographical and social isolation as well as stigma for help-seeking [27,32] are all candidate explanations for the significantly greater rate of suicide among adolescents and young adults in rural areas.

Older rural adults, especially men, are also among those at highest risk for suicide. There are more veterans in rural areas, with more than 25% of all veterans living in rural places [33], and older veterans who die by suicide are more likely to live in rural areas compared to their younger counterparts [34], and this may also be a contributing factor to the greater suicide rate. Other potential explanations of the mental health disparities among rural older populations focus on greater stigma for seeking help in older populations, higher social isolation for some rural elderly, and less perceived need for mental health services among rural veterans [35,36]. However, additional research that tests candidate mechanisms for why rural disparities exist for suicidal and mental health outcomes is needed.

### Race/Ethnicity

While individuals living in rural communities often experience worse mental health outcomes, research suggests that among people living in rural communities, racial and ethnic minorities experience substantial disparities that are often masked by viewing aggregated data [37]. More nuanced and specific data analyses suggest, however, that compared with rural non-Hispanic Whites, the prevalence of frequent mental distress is higher among rural American Indian/ANs (AI/AN) and rural African Americans, while depression is more common among rural AI/AN compared with rural non-Hispanic Whites [37].

When considering rural disparities in mental health treatment, the impact of race and ethnicity is considerable. For example, research suggests that racial and ethnic minorities living in rural areas are more likely to live in persistent poverty and be unemployed or underemployed [38,39]. In addition, while stigma related to mental disorders and seeking treatment for has been a well-documented factor associated with rural disparities, it is especially pronounced among racial and ethnic minorities [39,40], for whom issues of cultural mistrust are high [41–43] and the acceptability of psychological and psychiatric forms of treatment is low [44].

## A Research and Clinical Priority: Suicide

According to the Centers for Disease Control and Prevention, in 2017 suicide was the 10th leading cause of death overall in the USA [45]; however, there is a markedly higher risk of suicide among rural populations and thus an urgent need for translational research to inform culturally appropriate and specific prevention approaches.

Despite significant research around rural suicides, and the risk and protective factors associated with its increased incidence, additional studies are warranted that will improve our understanding of the underlying mechanisms and interactions. Demographically, in their updated review of the literature [46], Hirsch and Cukrowicz found that rurality may interact with other demographic risk factors such as gender, age, and race and ethnicity. Specifically, the authors found that deaths by suicide among rural individuals are generally higher among males, older individuals, and Latinos, in the USA [46]. Several other risk factors have been identified that are unique to rural areas: geographic isolation [46,47], factors associated with an agrarian lifestyle [46], access to lethal means such as firearms and pesticides [4,47], and a culture that promotes individualism and rugged independence [47,48] that may also promote stigma associated with mental illness or seeking treatment for suicidality.

The persistent problem of suicide, particularly among rural communities, points to the opportunity for researchers to use a research framework such as that developed by NIMHD, especially in collaboration with providers and members of the rural community to continue developing, implementing, and improving existing suicide prevention efforts that can appropriately address the unique and complex challenges and assets of rural areas. While innovative approaches in early suicide risk detection and prevention are advancing the field of suicide prevention, they do not address the disparities that continue to affect rural populations (and many underserved populations), if they are not reaching them. Therefore, to maximize impact, researchers investigating rural mental health should consider multiple domains and levels of influence in their study designs and address the complexities of multidimensional risks and protective factors in rural life.

## Conclusion: Challenges and Opportunities

Rural residents in the USA disproportionately suffer the negative effects of living with unmet or under-met mental health needs. The nature of this long-standing problem is well characterized. What is needed are research studies that test hypotheses about causality at multiple levels of influence and domains, consistent with the NIMH mechanism-focused, experimental therapeutics approach, especially using contemporary methods (e.g., SMART, stepped wedge designs, multilevel analysis, and MOST) [49,50]. Research that explores innovative care models for rural communities, tests suicide prevention strategies, and promotes improved access to mental health providers including non-specialty care practitioners will help make the desired impacts on rural mental health. Research that uses novel approaches to address stigma reduction to explicitly test the impact on behavior (e.g. help-seeking, treatment engagement or adherence, provider interactions, etc.) and patient outcomes is also needed. The NIMHD framework provides a useful structure to guide study designs that can address the complexities of delivering rural mental health care and to identify mechanisms underlying disparity as well as how best to remedy them.
